# Insights into mechanisms of ubiquitin ADP-ribosylation reversal

**DOI:** 10.1042/BST20240896

**Published:** 2024-11-25

**Authors:** Zhengrui Zhang, Chittaranjan Das

**Affiliations:** Department of Chemistry, Purdue University, West Lafayette, IN 47907, U.S.A

**Keywords:** ADP-ribosylation, macrodomain, molecular mechanisms, ubiquitins

## Abstract

Ubiquitination and ADP-ribosylation are two types of post-translational modification (PTM) involved in regulating various cellular activities. In a striking example of direct interplay between ubiquitination and ADP-ribosylation, the bacterial pathogen *Legionella pneumophila* uses its SidE family of secreted effectors to catalyze an NAD^+^-dependent phosphoribosyl ubiquitination of host substrates in a process involving the intermediary formation of ADP-ribosylated ubiquitin (ADPR-Ub). This noncanonical ubiquitination pathway is finely regulated by multiple *Legionella* effectors to ensure a balanced host subjugation. Among the various regulatory effectors, the macrodomain effector MavL has been recently shown to reverse the Ub ADP-ribosylation and regenerate intact Ub. Here, we briefly outline emerging knowledge on ubiquitination and ADP-ribosylation and tap into cases of direct cross-talk between these two PTMs. The chemistry of ADP-ribose in the context of the PTM and the reversal mechanisms of ADP-ribosylation are then highlighted. Lastly, focusing on recent structural studies on the MavL-mediated reversal of Ub ADP-ribosylation, we strive to deduce distinct mechanisms regarding the catalysis and product release of this reaction.

## Ubiquitination

**Figure 1.**
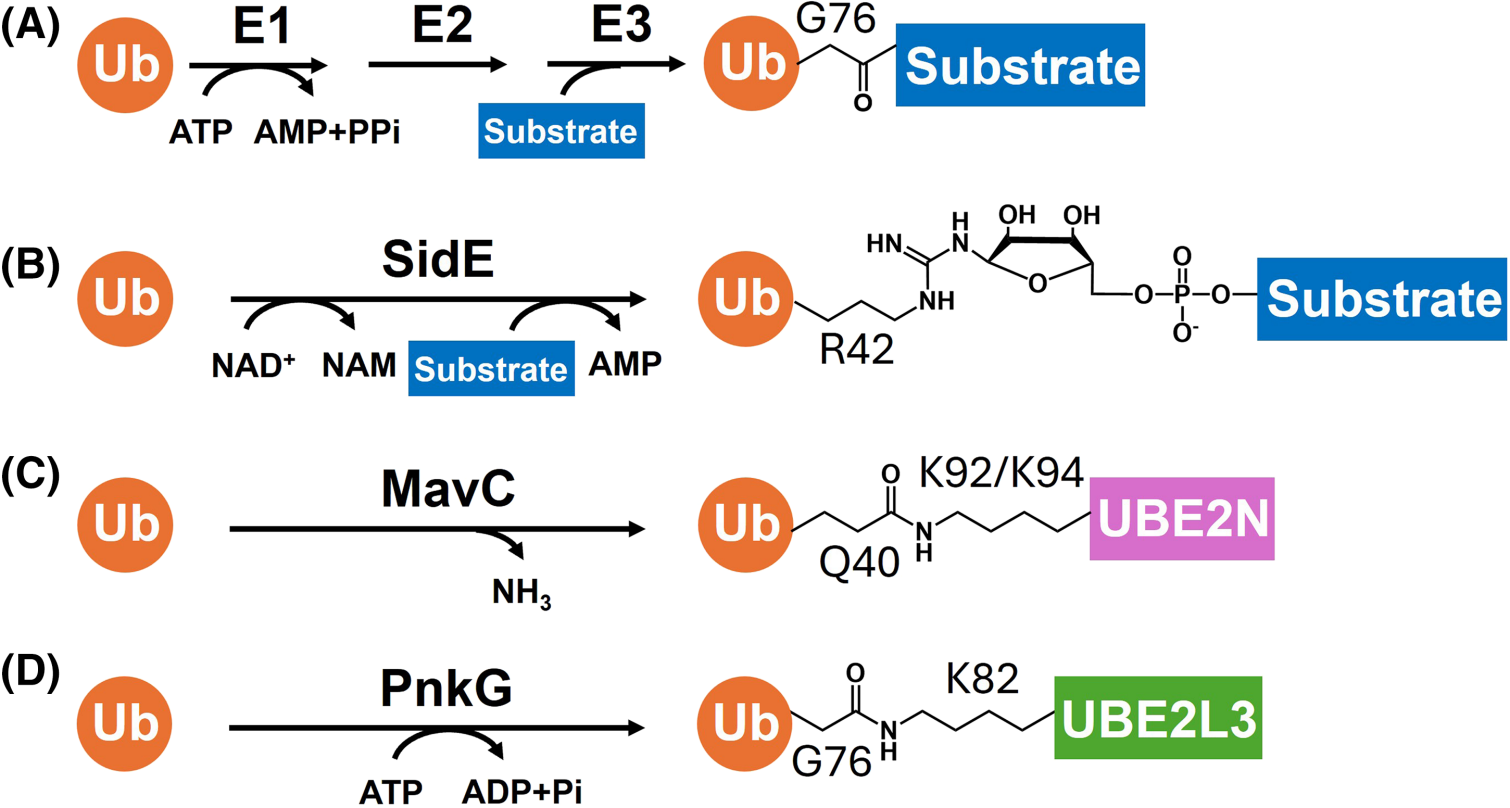
Different ubiquitination modes. (**A**) The canonical ubiquitination of eukaryotes. The G76 of Ub is activated by the E1 enzyme with ATP. Upon further transfer by E2 and E3 enzymes, Ub is attached to the targets forming amide or ester bonds. (**B**) SidE-mediated ubiquitination. R42 of Ub is ADP-ribosylated by the mono(ADP-ribosyl)transferase domain of SidE and further processed by the phosphodiesterase domain in the same enzyme to generate a phosphoribosyl linkage between hydroxyl residues of host targets and R42 of Ub. (NAM: Nicotinamide) (**C**) MavC-mediated ubiquitination. The *Legionella* effector MavC binds to UBE2N∼Ub complex and catalyzes transglutamination between Ub Q40 and UBE2N/Ubc13 K92 (or K94), forming an isopeptide linkage. (**D**) PnkG-mediated ubiquitination. The *Mycobacterium tuberculosis* effector PnkG catalyzes an ATP-dependent ubiquitination on UBE2L3/UbcH7 K82, with ATP hydrolysis on the γ-phosphate.

Ubiquitination is a post-translational modification (PTM) in which the 76 amino-acid eukaryotic protein ubiquitin (Ub) is covalently attached to cellular targets [1,2]. Canonical ubiquitination requires an ATP-dependent, E1-E2-E3 three-enzyme transfer cascade, with an amide bond forming between Ub G76 and the lysine (or first methionine) residue of the protein target or Ub itself (to assemble poly-Ub chains) (Figure 1A). Aside from the more commonly used amide-linked ubiquitination, non-lysine ubiquitinations occurring on either cysteine, serine, or threonine residues have also been characterized [3,4], indicating the intricacy of this PTM. Although proteinaceous substrates are widely known to be the recipients of ubiquitination, recent studies have shown that bacterial lipopolysaccharides, unbranched glucosaccharides, organelle phospholipids, and ADP-ribosyl moieties can also be ubiquitinated [5–10], suggesting that the substrate landscape of ubiquitination can be more expansive than currently known. Interestingly, bacterial effectors can mediate ubiquitination events that are distinct from the canonical one in eukaryotic systems. Specifically, the SidE effector family belonging to the Legionnaires’ disease-causing pathogen, *Legionella pneumophila*, can catalyze an NAD^+^-dependent two-step ubiquitination on various host targets, in which a phosphoribosyl linker is formed between Arg42 of Ub and residue hydroxyl groups of host targets [11–15] (Figure 1B). Another *L. pneumophila* effector MavC mediates transglutamination between Q40 of Ub and K92 (or, to a lesser extent, K94) of a host E2 enzyme, UBE2N (Ubc13), resulting in the formation of an isopeptide linkage [16] (Figure 1C). The *Mycobacterium tuberculosis* effector PknG single-handedly catalyzes an ATP-dependent ubiquitination reaction linking Ub G76 to K82 of the eukaryotic E2 enzyme UBE2L3 (UbcH7), with the concomitant hydrolysis of ATP at the γ-phosphate position [17] (Figure 1D).

The reversal of ubiquitination is catalyzed by deubiquitinases (DUBs), which restore ubiquitinated substrates to their unmodified form, thereby modulating cellular pathways involving ubiquitination [18,19]. In most cases, DUBs are peptidases that cleave the amide bond between G76 of Ub and the modified substrates (or another Ub as in poly-Ub chains). Recently, DUBs with additional esterase activities have also been profiled, suggesting their roles in the reversal of non-lysine ubiquitination [20]. In addition, specific DUBs have been characterized to function on unique types of ubiquitination. For example, ubiquitination on bacterial lipopolysaccharides and maltoheptaose can be reversed by a bacterial DUB functioning as an esterase [21]. SidE-mediated phosphoribosyl ubiquitination is reversed by two *L. pneumophila* phosphodiesterase effectors, DupA and DupB, with DUB-like function to release the host proteins from their ubiquitinated form [22,23]. The activity of MavC is counteracted by its paralog MvcA, which cleaves the isopeptide linkage to remove Ub from UBE2N [24].

## ADP-ribosylation

**Figure 2.**
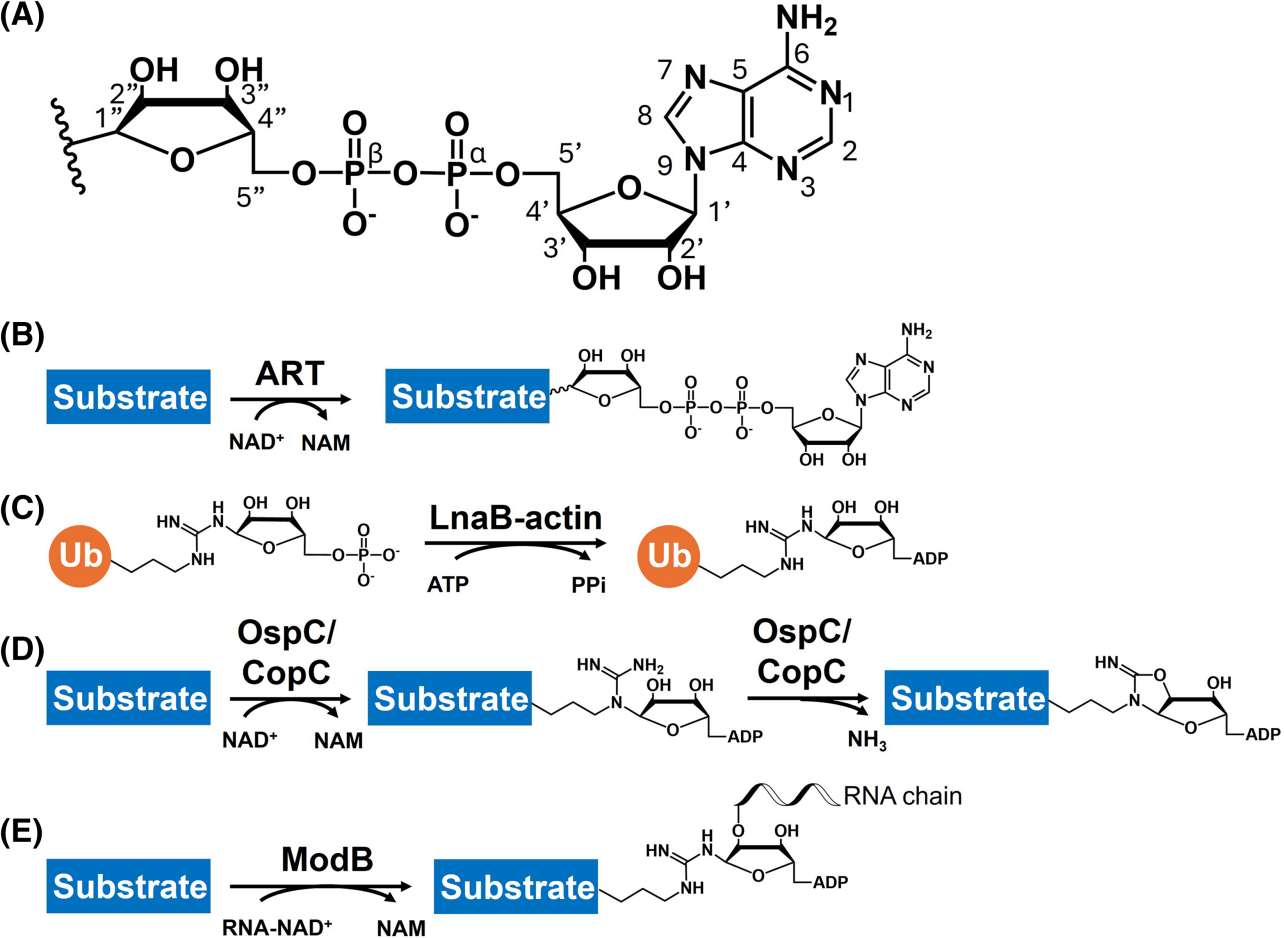
ADP-ribosylation and ADP-ribosylation-like processes. (**A**) Overall structure and atom numbering of ADP-ribose. (**B**) Canonical ADP-ribosylation process. ADP-ribose from NAD^+^ is transferred by ARTs or Sirtuins onto the substrate, forming *N*-, *O*-, or *S*- glycosidic bond. (**C**) ADP-ribosylation by LnaB-mediated AMPylation. The actin-dependent *Legionella* effector LnaB AMPylates the phosphate group of phosphoribosylated Ub, producing ADPR-Ub. (**D**) OspC/CopC-mediated ADP-riboxanation. The *S. flexneri* OspC family and the *C. violaceum* CopC mediate ADP-riboxanation on arginine residues of host targets. Structural studies reveal that this modification occurs in a stepwise manner, with ADP-ribosylation on arginine Nδ followed by a deamidative cyclization. (**E**) ModB-mediated RNAylation. The viral (ADP-ribosyl)transferase ModB catalyzes the transfer of RNA chain onto the arginine of host ribosomal proteins rS1 and rL2 following a mechanism akin to that of canonical ADP-ribosylation.

ADP-ribosylation refers to the addition of ADP-ribose (ADPR) (Figure 2A) from the nicotinamide adenosine diphosphate (NAD^+^) donor onto diverse cellular targets [25] (Figure 2B), including proteins, nucleotides, and small molecules. Enzymes catalyzing ADP-ribosylation belong to two major protein superfamilies, namely (ADP-ribosyl)transferases (ARTs) and Sirtuins [25–27]. Depending on the active site sequence motifs and the catalytic properties, ARTs are further classified into the ARTs diphtheria toxin like (ARTDs) and the ARTs cholera toxin like (ARTCs) subclasses [26]. In humans, ARTDs and ARTCs have alternative nomenclatures as poly-ADP-ribosyl polymerases (PARPs) and ecto-ARTs, respectively. Although NAD^+^, in most cases, serves as the direct ADPR donor for ADP-ribosylation, recent studies have revealed an indirect ADP-ribosylation process resulting from an unusual phosphoryl-AMPylation reaction catalyzed by the *Legionella* effector LnaB, which converts phosphoribosylated Ub (PR-Ub) to ADP-ribosylated Ub (ADPR-Ub) [28,29] (Figure 2C), indicating that ADP-ribosylation can be generated by other means. Of note, previous studies have characterized phosphodiesterases that generate phosphoribosylated proteins with unclear functional outcomes [30–32]. With the discovery of LnaB, it is tempting to speculate that these phosphoribosyl modifications can be further processed to generate ADP-ribosylations by unknown AMPylases. However, whether this is a widespread biochemical reaction in other organisms or a specific pathway during *Legionella* infection awaits further investigation.

In addition to ADP-ribosylation, ARTs can be involved in ADP-ribosylation-like reactions. For instance, the OspC family (from *Shigella flexneri*) and CopC (from *Chromobacterium violaceum*), can catalyze calmodulin-dependent ADP-riboxanations on arginine residues of different host targets, thereby manipulating host immune responses and the translational machinery [33–35]. Recent structural studies suggest that this reaction likely occurs in a stepwise fashion [36,37], with ADP-ribosylation first occurring on the Nδ atom of arginine, instead of Nω as in common arginine ADP-ribosylation events. A conserved aspartate residue near the active site then mediates the subsequent deamidative cyclization (Figure 2D). Another ADP-ribosylation-related modification is exemplified by the viral ART ModB, which accepts NAD^+^-capped RNA chain as substrate and attaches the RNA chain onto arginine residues of ribosomal proteins rS1 and rL2 [38] (Figure 2E), leading to potential host translational manipulation.

## Cross-talk between ubiquitination and ADP-ribosylation

**Figure 3.**
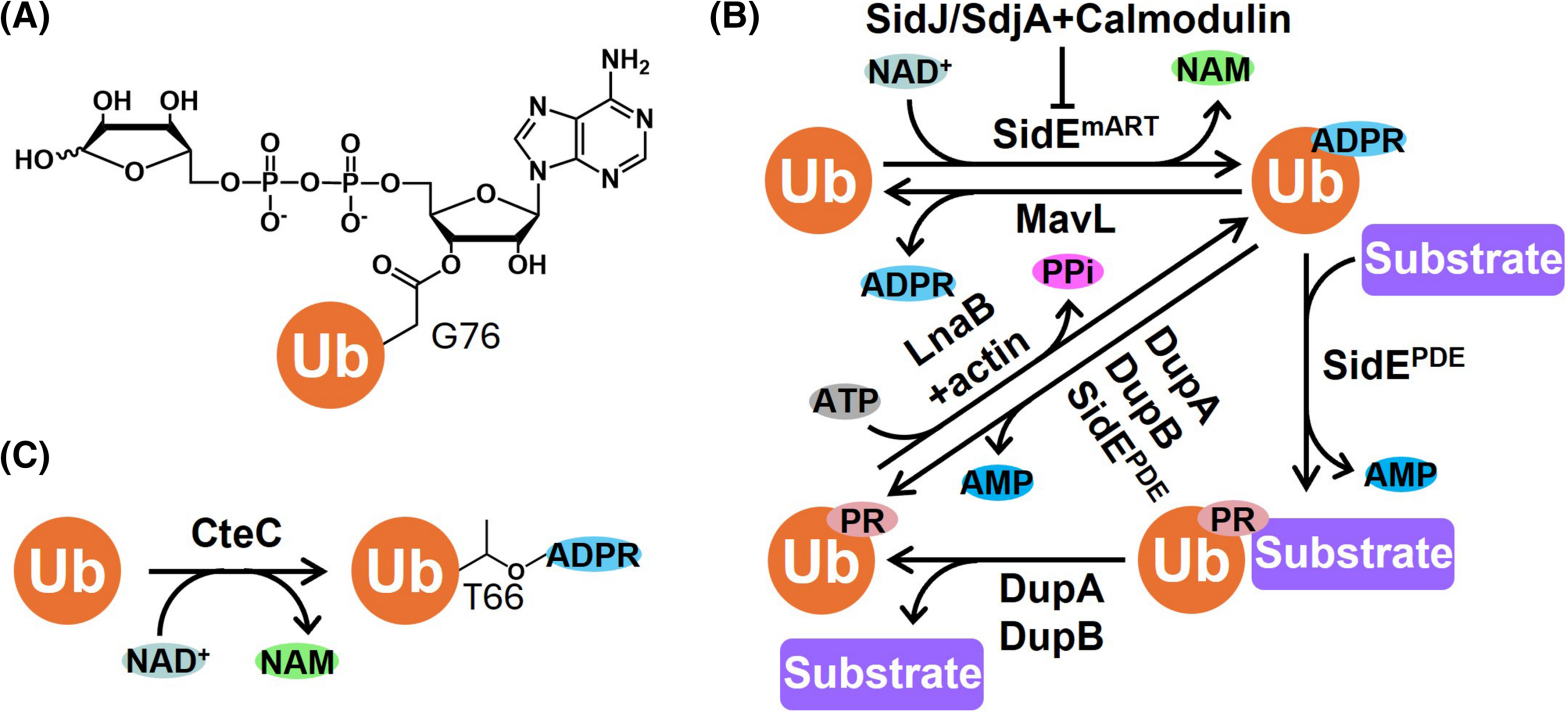
Cross-talk between ubiquitination and ADP-ribosylation. (**A**) Product of PARP9/DTX E3s-catalyzed ADPR ubiquitination, with an ester bond formation between Ub G76 and O3′ of ADPR. (**B**) CteC-mediated Ub ADP-ribosylation. The *C. violaceum* effector CteC catalyzes mono(ADP-ribosyl)ation on Ub T66, resulting in a general blocking of host ubiquitin signaling. (**C**) SidE-mediated ubiquitination and its regulation. Ub is first ADP-ribosylated on R42 by the mono-ART domain of SidE, forming ADPR-Ub, which is then processed by the phosphodiesterase domain of SidE, resulting in a phosphoribosyl linker between Ub R42 and the substrate. DupA and DupB reverse the ubiquitination by removing the PR-Ub from the substrate. The resulting PR-Ub can be AMPylated by the actin-dependent effector LnaB to regenerate ADPR-Ub. The ADP-ribosylation can then be reversed by MavL to recover intact Ub. Additionally, SidJ and SdjA, upon activation by calmodulin, polyglutamylate the active site glutamate residue to inactivate the SidE mART domain.

Although ubiquitination and ADP-ribosylation have primarily been looked at as PTMs involved in regulating various cellular activities, direct cross-talks between ubiquitination and ADP-ribosylation are emerging as new mechanisms converging on novel forms of PTMs. An inherent interplay in humans was first observed in the case of PARP9 and DTX family E3 ligase DTX3L, which is originally believed to ADP-ribosylate the C-terminus of Ub to prevent canonical ubiquitination and to play a role in DNA repair [39]. It was later shown that DTX3L alone is sufficient to catalyze this reaction [40]. Strikingly, recent studies showed that not only DTX3L, but other members of the DTX family mediate the transfer of ubiquitin onto the O3′ of ADPR [8–10] (Figure 3A), indicating that this reaction should be more suitably described as ‘ubiquitination of ADPR’ rather than ‘ADP-ribosylation of Ub’. This unique ubiquitination event can occur on both ADP-ribosylated protein and nucleic acid substrates [8,9]. However, the exact functions of this type of ubiquitination have not been firmly demonstrated.

Two other examples of direct intercommunion between ubiquitination and ADP-ribosylation are observed during bacterial infections. The *C. violaceum* effector CteC is an ARTC-type mono(ADP-ribosyl)tranferase that mono(ADP-ribosyl)ates Ub (or Ub chains) on T66 (Figure 3B), resulting in a blockade of overall host ubiquitination signaling [41]. Another pathogen-mediated Ub ADP-ribosylation event, as mentioned before, is observed in an NAD^+^-dependent noncanonical ubiquitination mediated by *L. pneumophila*. Specifically, the *Legionella* SidE family first ADP-ribosylates Ub R42, forming ADPR-Ub as an intermediate, which is used to ubiquitinate host targets via a phosphoribosyl linker in concert with the catalytic function of the phosphodiesterase domain in the same effector family [11–13] (Figure 3C). Like CteC-mediated Ub ADP-ribosylation, modification of Ub by SidE family can also happen on Ub chains [42]. Diverse targets of this NAD^+^-dependent ubiquitination have been discovered, including ER-associated Rab GTPases, reticulon 4, and the host DUB USP14 [11–13,22,23,43]. Phosphoribosyl ubiquitination on these targets leads to the interference of host ER and Golgi dynamic and promotes *Legionella* infectivity. Recent studies showed that this atypical ubiquitination pathway is fully reversible and finely regulated by other *Legionella* effectors (Figure 3C). The phosphodiesterase effectors DupA and DupB cleave PR-Ub off the substrates [22,23], thus releasing the host substrates in their unmodified form. The resulting PR-Ub can be processed by the actin-dependent phosphoryl-AMPylase LnaB to produce ADPR-Ub [28,29], and the ADPR moiety is subsequently removed by the (ADP-ribosyl)hydrolase effector MavL to regenerate intact Ub [44]. Additionally, two other effectors, SidJ and SdjA, activated by host calmodulin, catalyze poly-glutamylation on the ART domain of the SidE family, thus shutting off the noncanonical ubiquitination pathway [45–51].

## Chemistry of ADPR and ADP-ribosylation

**Figure 4.**
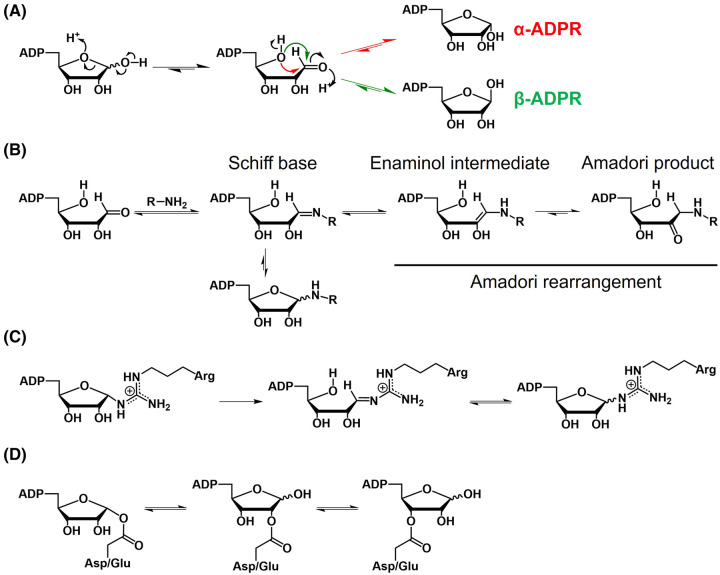
Chemistry of ADPR and ADP-ribosylated residues. (**A**) Spontaneous equilibrium of free ADPR between α- and β-anomers through a reactive open-ring aldehyde. (**B**) The reactive aldehyde can react with free amine to form a Schiff base, which can then undergo Amadori rearrangement to form the keto amine product. (**C**) ADPR attached to arginine residue exhibits an equilibrium between anomers. (**D**) Acyl migration in ADP-ribosylated aspartate/glutamate.

Depending on the residue or molecule ADPR is added onto, *N*-, *O*-, or *S*-glycosidic bond can form between C1″ of ADPR and the receiving molecule [25]. In the case of poly(ADP-ribosyl)ation (catalyzed by ARTDs or PARPs), the glycosidic bond is formed between C1″ of the distal ADPR and O2′ or O2″ of the proximal ADPR [52] (Figure 2A). The ADPR moiety alone (generated from NAD^+^ hydrolysis or ADP-ribosyl hydrolysis) displays a spontaneous in-solution equilibrium between the open-chain aldehyde form and the closed-ring α- and β-ribofuranose anomers [53] (Figure 4A). The aldehyde form is a reactive species that can participate in biochemical reactions [54]. For example, a free amine can react with the open-chain aldehyde to form a Schiff base, which can then undergo Amadori rearrangement to form a keto amine product (Figure 4B). Such products have been captured in certain cases where they enlighten the catalytic mechanisms of the (ADP-ribosyl)hydrolases [55,56]. ADP-ribosylation on certain protein residues can also display unique spontaneous chemistry. Specifically, arginine ADP-ribosylation switches between α- and β-anomers in solution [57] (Figure 4C) whereas *O*-acetyl-ADPR and aspartate/glutamate ADP-ribosylation undergo acyl migration among three hydroxyl groups of the distal ribose [58–61] (Figure 4D). These spontaneous, nonenzymatic rearrangements suggest the complexity of ADP-ribosylation, posing challenges for mechanistic studies on ADP-ribosylation-related processes, especially its reversal.

## Enzymes catalyzing ADP-ribosylation reversal

The reversal of ADP-ribosylation is catalyzed by (ADP-ribosyl)hydrolases containing three families of enzymes, (ADP-ribosyl) hydrolases (ARHs), macrodomains, and ‘NAD- and ADP-ribose’-associated (NADAR) enzymes [62,63]. ARHs are divalent ion-dependent metalloenzymes first discovered in the phototrophic bacterium *Rhodospirillum rubrum* as a regulator in nitrogen fixation. Specifically, the dinitrogenase reductase-activating glycohydrolase (DraG) reverses the arginine mono(ADP-ribosyl)ation on dinitrogenase reductase, therefore restoring its enzymatic activity [64,65]. In humans, the DraG equivalent ARH is ARH1, which specifically reverses arginine mono(ADP-ribosyl)ation [66]. In addition, another human ARH, ARH3, hydrolyzes *O*-glycosidic linkages including serine mono(ADP-ribosyl)ation [67], *O*-acetyl-ADPR [68], and poly(ADP-ribosyl)ation [69]. Macrodomains, on the other hand, do not require metal ions for their catalysis and have a wider substrate scope. Historically, three macrodomain subclasses have been identified, namely MacroD-type, PARG-like, and ALC1-like [70]. MarcoD-type macrodomains mainly hydrolyze glutamate or aspartate mono(ADP-ribosyl)ation [71,72], whereas PARG-like macrodomains cleave poly-ADPR chains [73]. ALC1-like macrodomain are exemplified by DarG (DNA ADP-ribosyl glycohydrolase) in bacteria and TARG1 in humans. DarG reverses mono(ADP-ribosyl)ation on thymidine of single-stranded DNA (ssDNA) [74], whereas TARG1 can act on a broad range of mono(ADP-ribosyl)ated substrates [55,75–78]. A recent study by our group uncovered a new macrodomain class previously termed DUF4804, represented by the *Legionella pneumophila* effector MavL, to be specific towards arginine mono(ADP-ribosyl)ation [44]. The NADAR family, related to *Escherichia coli N*-glycosidase YbiA, has been recently characterized to reverse mono(ADP-ribosyl)ation on guanosine of ssDNA [63].

## Mechanistic investigations on ADP-ribosylation reversal

To date, one of the most straightforward and informative approaches to studying enzyme mechanisms of ADP-ribosyl hydrolysis is through structural biology means by obtaining the complex structure of ADPR-bound (ADP-ribosyl)hydrolases. In high-resolution co-crystal structures, water molecules can often be unambiguously observed near the active site, thus providing valuable information for deducing the putative catalytic process. In rare examples, the active-site water network and orientation of putative catalytic waters can be precisely determined by neutron crystallography [79]. Of note, it is also important to keep in mind that, although ADPR is the catalytic product, structures of ADPR-bound (ADP-ribosyl)hydrolases may not necessarily represent the exact stereochemistry of the product-bound state due to the aforementioned spontaneous anomerization of ADPR. Therefore, additional experimental evidence should be taken into consideration for proposing catalytic mechanisms, rather than relying solely on the ADPR-bound structures.

**Figure 5.**
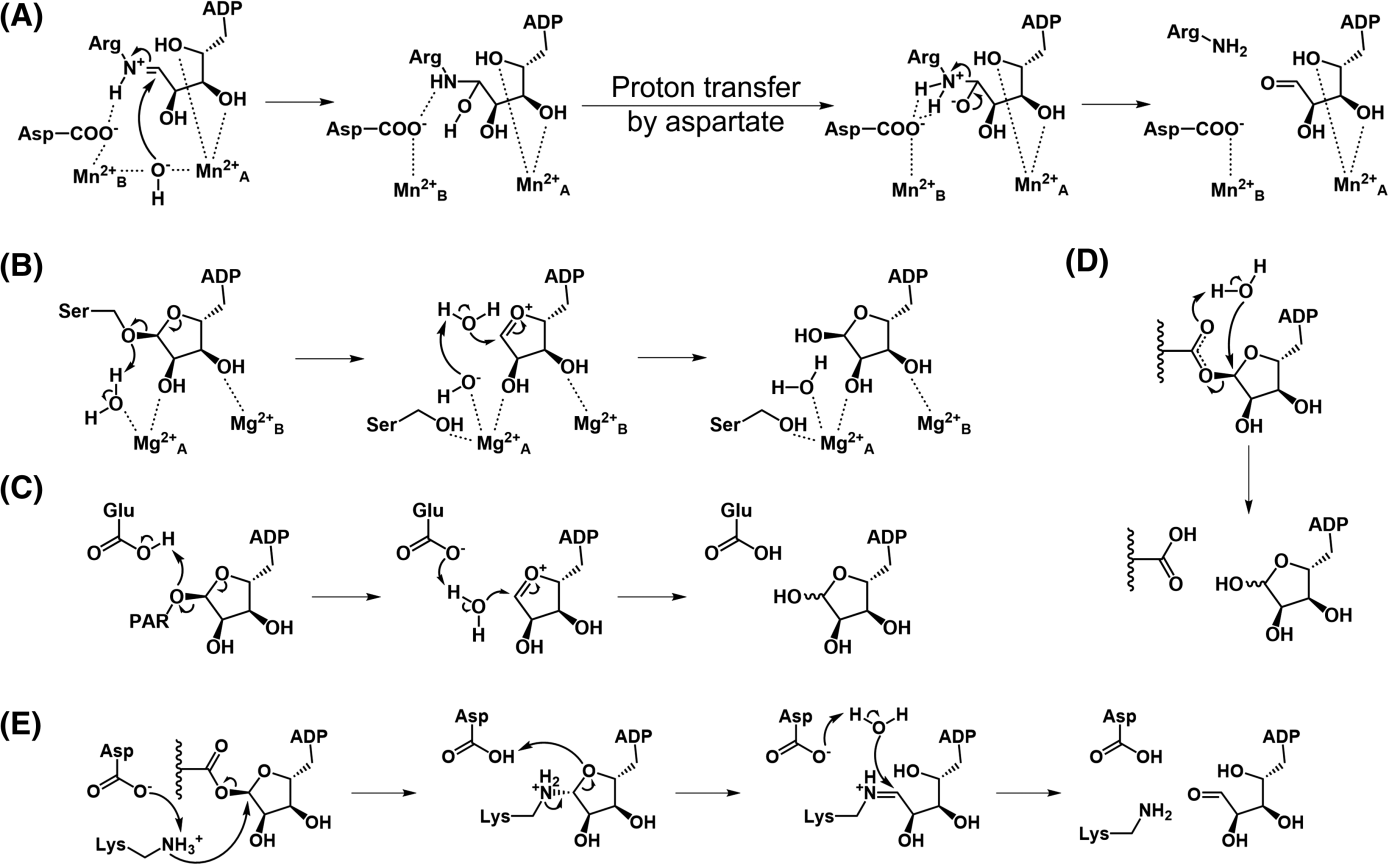
Proposed mechanisms of ADP-ribosyl hydrolysis by different (ADP-ribosyl)hydrolases. (**A**) ADP-ribosyl hydrolysis by DraG. The hydroxide ion is held by the divalent metal ions and performs nucleophilic attack on C1′ of open-ring ADPR-arginine, with a nearby aspartate mediating the proton transfer of the reaction. (**B**) ADP-ribosyl hydrolysis by ARH1. The presence of an oxocarbenium ion intermediate was confirmed based on a solvoytic methanol-based experiment. (**C**) ADP-ribosyl hydrolysis by PARG-like macrodomains. An S_N_1-type mechanism with the formation of an oxocarbenium ion intermediate was proposed, and a conserved glutamate residue is present to activate the catalytic water molecule. PAR: poly-ADPR. (**D**) ADP-ribosyl hydrolysis by MarcoD-type macrodomains. A substrate-assisted S_N_2-type mechanism with the carbonyl oxygen of the ester bond activating the catalytic water. (**E**) ADP-ribosyl hydrolysis by ALC1-like macrodomains. The presence of an Amadori ADPR intermediate is proposed based on structural studies, with a nearby glutamate acting as the general base to activate the water molecule. However, the glutamate residue is not conserved across the ALC1-like macrodomains, indicating that alternative mechanisms may be employed by other members in this macrodomain class.

For the ARH family, the structure of the ADPR-bound bacterial DraG was first characterized, in which an Amadori ADPR product was found to be attached to a lysine from the nearby DraG protomer [56]. The authors therefore proposed an ADP-ribosyl hydrolysis mechanism involving an open-ring Amadori intermediate [56] (Figure 5A), which is in line with the anomeric properties of mono(ADP-ribosyl)ation on arginine residues (Figure 4C). The human homolog of DraG, ARH1, has also been crystallized with different metal coordinating forms with ADPR or an ADPR analog [62]. However, these structures do not provide sufficient information for a clear catalytic mechanism. Despite this obscurity, ARH1 does not have a DraG-specific aspartate (Figure 5A), indicating that it likely adopts a distinct mechanism. The human ARH3 has been structurally characterized in multiple states bound to different substrates/ADPR/ADPR analogs [62,80]. Using a catalytic Mg^2+^ ion, it follows a distinct mechanism involving an oxocarbenium ion intermediate, which has been confirmed using solvolytic trapping by methanol [80] (Figure 5B).

The mechanisms of macrodomain (ADP-ribosyl)hydrolases are more extensively studied. For PARG-like macrodomains, the binding of the poly-ADPR has been shown to increase the p*K*_a_ of the catalytic glutamate, which allows it to protonate the leaving group (poly-ADPR) with the formation of an oxocarbenium intermediate. The same glutamate residue can then act as a general base to deprotonate the catalytic water to attack the intermediate and form free ADPR [81–85] (Figure 5C). Depending on the side chain orientation of the catalytic glutamate and the local structure of the active site, the ADPR anomer formed can vary [81–83,85,86]. In contrast, examples of viral MacroD-type macrodomains lacking the active site aspartate/glutamate residue indicate that this class likely adopts a mechanism independent of aspartate/glutamate [79,87–90]. Additionally, the observation of a putative catalytic water near the C1″ [71,91,92] as well as several MacroD structures in complex with β-ADPR [87–94] point to a possible substrate-assisted S_N_2 type of mechanism. Mutagenesis designed to displace this catalytic water effectively reduces the enzymatic activity [92], thus validating the role of the catalytic water. In this mechanism, the catalytic water is activated by the ester bond of the substrate and performs nucleophilic attack to the C1″ center [86] (Figure 5D). ALC1-like macrodomains are comparatively less studied and the mechanism appears to vary within this class. The available structure of TARG1, in complex with ADPR, reveals an Amadori ADPR product linked to the catalytically indispensable lysine, indicating an intermediate of this form [55]. In addition, an aspartate residue is also found near the distal ribose region, which is believed to activate the catalytic water molecule [55,86] (Figure 5E). However, DarG, another ALC1-like macrodomain, only harbors the catalytically essential lysine but lacks the aspartate [74], therefore it is not yet clear in this case how the water can be activated, perhaps through a yet-unknown substrate-assisted route. The mechanism of the recently discovered MavL-like macrodomains will be discussed in detail in the next section.

The NADAR enzymes were discovered and characterized recently, with only one α-ADPR-bound structure determined so far. In this structure, a conserved glutamate and a lysine near the distal ribose are observed [63], hinting that this (ADP-ribosyl)hydrolase family may adopt a mechanism resembling that of TARG1.

## Reversal of Ub ADP-ribosylation by MavL

**Figure 6.**
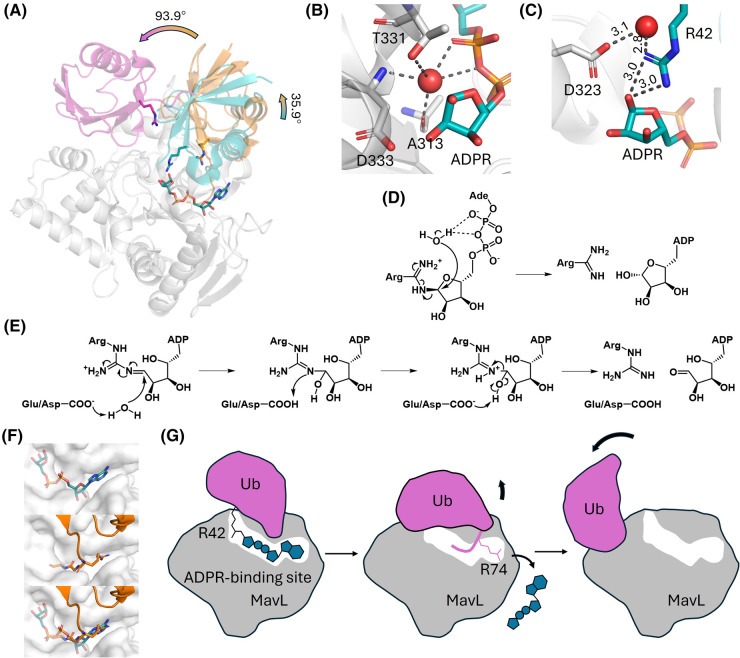
Mechanism of Ub ADP-ribosyl hydrolysis by MavL. (**A**) Superposition of three Ub-bound MavL structures. Grey: MavL; Teal: Ub observed in ADPR:Ub:MavL structure (PDB:8IPJ); Orange: Ub observed in Ub:MavL structure (PDB:8XEP); Magenta: Ub observed in MavL-UbVME structure (PDB:8DMQ). The relative Ub movements between structures are marked, with Ub R42 and ADPR-binding site shown as sticks. (**B**) A phosphate-coordinating water molecule was observed in ADPR-bound MavL structure (PDB:8DMR). This water molecule is located underneath the distal ribose and held by A313 backbone carbonyl, T331 hydroxyl group, D333 backbone nitrogen, and the ADPR di-phosphate. (**C**) A water molecule held by Ub R42 and MavL D323 is observed in the ADPR:Ub:MavL structure. Crucial distances between atoms are marked. (**D**) A possible substrate-assisted S_N_2-type mechanism employed by MavL-like macrodomains based on (**B**). The α-phosphate functions as a general base to activate the water molecule, forming a hydroxide ion that attacks C1″. β-ADPR is generated as the product. (**E**) A possible mechanism involving an open-ring ADPR-arginine intermediate is proposed based on (**C**). D323 of MavL is conserved across MavL-like macrodomains as either aspartate or glutamate. This residue possibly functions to activate the catalytic water and facilitate the release of the product. (**F**) Clashes between ADPR and Ub C-terminus at the ADPR-binding site in MavL. Top: ADPR-binding site in ADPR-bound MavL structure. Middle: C-terminus (R74-G75-G76) of Ub in Ub:MavL structure. Bottom: Superposition of ADPR-bound MavL and Ub:MavL structures suggests that the Ub C-terminus may intrude the ADPR-binding site later in the catalysis. (**G**) Proposed product release mechanism in MavL-mediated Ub ADP-ribosylation reversal. Step 1: ADPR-Ub binds to MavL at the active site and is hydrolyzed. Step 2: Ub undergoes a slight tilt and inserts its C-terminus into the ADPR-binding site of MavL, thus displacing the free ADPR off the active site. Step 3: Ub undergoes further movement and dissociates from MavL, exposing the MavL active site for future catalysis.

The reversal of Ub ADP-ribosylation by MavL suggests that arginine mono(ADP-ribosyl)ation can be reversed by macrodomains, as also observed in the case of another *Legionella* macrodomain effector Larg1 that reverses arginine mono(ADP-ribosyl)ation on mitochondrial ADP/ATP translocases [95,96]. Structures of MavL, in complex with ADPR, Ub, or ADPR-Ub, have been determined (Figure 6A) to provide insights into the catalytic mechanism [28,29,44]. The structure of ADPR-bound MavL reveals a phosphate-coordinating water to be the putative catalytic water [44] (Figure 6B). Same phosphate-coordinating water molecules can be observed in structures of Larg1 and two *Drosophila* MavL-like macrodomains [44,95,96], indicating that this macrodomain family likely adopts similar ADP-ribosyl hydrolysis mechanisms. In MavL, this water is coordinated by the di-phosphate group of ADPR and A313, T331, and D333 residues of the enzyme (Figure 6B). During the catalysis, the α-phosphate of the substrate ADPR moiety first functions as a general base to deprotonate the water molecule, which subsequently performs nucleophilic attack to C1″ to produce β-ADPR (Figure 6D). A similar substrate-assisted S_N_2-type mechanism has also been proposed for some MacroD-type macrodomains [71,79], yet the low p*K*_a_ of phosphate is presumed to disfavor this proposition [85]. Inspection of the water coordination site complemented with mutagenesis study, however, does not reveal any other moieties that can function as the general base [44]; one possibility is that binding to MavL may alter the p*K*_a_ of the phosphate in ADPR allowing it to activate the water. Strikingly, the MavL structure in complex with the substrate ADPR-Ub but in a cleaved state hinted another putative mechanism [29]. In this structure, the O1″ of α-ADPR is 3.0 Å away from the Nω of Ub R42 (Figure 6C), suggesting the possible formation of an open-ring Amadori ADPR intermediate comparable to the one proposed during the catalysis of DraG (Figure 5A). In support of this, a conserved negatively charged residue (D323 in MavL) can be found nearby, which could function as the general base to activate an adjacent water molecule (Figure 6C). In this mechanism, D323 activates the catalytic water, mediates proton transfer onto the Nω of the arginine, and facilitates the release of arginine (Figure 6E). Although this mechanism appears valid, the lack of evidence showing the open-ring ADPR intermediate renders this mechanism mostly hypothetical at this stage.

Apart from the distinct ADP-ribosyl hydrolysis mechanism, MavL also adopts interesting Ub recognition features as selectivity determinants for protein substrates. So far, three Ub-bound MavL structures have been determined, yet all of them display unique binding interfaces. The ADPR:Ub:MavL structure (complex with the cleaved substrate) mentioned before reveals a Ub binding site most likely representing a catalytically poised state [29], with the Ub R42 in proximity to the ADPR distal ribose (Figure 6A,C). In comparison, the Ub:MavL structure captured in absence of the bound nucleotide presents a slightly tilted Ub with an angle of 36° (Figure 6A), in which the C-terminus of Ub (R74-G75-G76) inserts itself into the ADPR-binding site [28] (Figure 6F), hinting a possible post-catalytic ADPR release mechanism by Ub C-terminus displacement. In the third Ub-bound MavL structure, captured as a MavL-UbVME covalent adduct [44], a completely unique Ub binding site is observed, with an additional 94° Ub swing compared with the Ub:MavL structure (Figure 6A). The drastic difference regarding Ub orientations relative to MavL can be simply attributed to (1) the likely flexible motion of Ub, as suggested by the weak interaction between Ub and MavL, with a *K*_d_ of ∼100 µM [29,44], and (2) the crystallographic packing effect, as observed in both Ub:MavL and MavL-UbVME structures that Ub makes contacts with two MavL protomers within the asymmetric unit [28,44]. Nevertheless, should the interfaces observed in these structures be physiologically relevant, a stepwise product release mechanism can be postulated (Figure 6G): ADPR-Ub first binds to the active site of MavL and is hydrolyzed. A slight rotational movement of Ub then assists ADPR release through the insertion of its C-terminus into the ADPR-binding site. Lastly, Ub undergoes a further swinging movement to dissociate from MavL and expose the active site for future catalysis. The exact product release mechanism of this reaction, however, still requires further investigation.

## Future directions

Currently, mechanistic investigations of ADP-ribosylation reversal rely mainly on structural studies complemented by carefully designed biochemical assays. Yet, the convoluted ADPR chemistry (Figure 4) and the difficulty in capturing reaction intermediates pose great challenges in deciphering the precise catalytic mechanisms. On top of that, the contribution of the substrate being ADP-ribosylated is often overlooked in the mechanisms, as most studies in this field focus on the hydrolysis of the glycosidic bond on residue levels. Recent studies on MavL, however, disclose the importance of protein-level substrate recognition during catalysis, as mutation disrupting the MavL:Ub interface results in reduced activities [29,44]. Moreover, other MavL-like macrodomains exhibit rather weak activities towards ADPR-Ub, indicating that the substrate recognition is likely beyond just the arginine mono(ADP-ribosyl)ation [44]. Collectively, these discussions manifest the importance of obtaining comprehensive substrate scopes of (ADP-ribosyl)hydrolases and subsequent structural information in complex with, if any, the full substrates.

On another note, some ADP-ribosylation events appear to be irreversible. For example, Ub ADP-ribosylation on T66 by CteC cannot be reversed by known ADP-ribosyl hydrolases [41,44]. With recent discoveries of new ADP-ribosyl hydrolases, it is likely that some (ADP-ribosyl)hydrolases with unique specificity and sequence features remain to be revealed. Current discoveries of (ADP-ribosyl)hydrolases mostly employ bioinformatical approaches complemented by biochemical validation, however, approaches identifying (ADP-ribosyl)hydrolases at the proteome scale have not been reported. Development of a general method for this purpose, for example, a chemical probe that specifically modifies (ADP-ribosyl)hydrolases, would greatly enhance our knowledge in this field.

## Perspectives

- The recent characterizations of the Ub (ADP-ribosyl)hydrolase MavL reveal a pivotal step in the regulation of SidE-mediated noncanonical ubiquitination. Structural insights into this macrodomain effector shed light on the postulated mechanisms of ADP-ribosyl hydrolysis and Ub-assisted product release. Although the exact details of the catalytic process remain elusive, these studies provide a solid foundation for future characterization of this macrodomain class.
- The specificity of MavL-like macrodomains is beyond just the ADP-ribosylated residue, indicating that the substrate harboring ADP-ribosyl modification also contributes to the ADP-ribosyl hydrolysis process. This could be a common phenomenon for bacterial enzymes, as they usually target specific substrate(s). Future studies are needed to elucidate the precise substrate scope and protein-level recognition of (ADP-ribosyl)hydrolases.
- Recent discoveries of new (ADP-ribosyl)hydrolases indicate the existence of uncharacterized (ADP-ribosyl)hydrolases, especially given that some ADP-ribosylations have no known enzymes for reversal. Development of robust and universal approaches identifying new (ADP-ribosyl)hydrolases will assist better understanding of ADP-ribosylation-related cellular processes.

## Abbreviations

ADPR-Ub: ADP-ribosylated ubiquitin
DUB: deubiquitinase
NAD^+^: nicotinamide adenosine diphosphate
PARP: poly-ADP-ribosyl polymerase
PTM: post-translational modification
ssDNA: single-stranded DNA
